# The Treatment of Drunkards

**Published:** 1871-11

**Authors:** 


### The Treatment of Drunkards.

An article in a recent number of *Macmillan's Magazine* gives an interesting account of one of the asylums for drunkards of which several have been founded with great success in America. The American laws, more paternal than our own, recognize the necessity for guarding the habitual drunkard against himself, and do not leave him at large until drink has ended in crime. Thus we find that "the committee of an habitual drunkard, duly appointed under the provisions of the laws of the State, can place such habitual drunkard in the asylum, and authorizes his detention under such restraint as may be necessary to prevent his escape." A large proportion, however, of the inmates of these asylums are voluntary patients who place themselves there with a view to self-cure.

The New York State Inebriate Asylum has been in active operation since May, 1867. From that date to the end of 1868 the number of admissions was 310; 272 being paying patients, and 38 free. Of these patients, 147 were classed as periodical and 163 as constant drinkers, and the following were their antecedents: 96 had delirium tremens, 31 convulsions, and 73 other diseases; 101 were in good health, and 9 were supposed to have an hereditary tendency to intemperance. The average length of time each patient remained in the asylum was ninety-six days; 113 were reformed after a first trial; 11 relapsed, but were reformed after a second trial; whilst 4 relapsed a second time, but were reformed after a third visit; 25 were discharged hopeless; and of the remainder, in 68 the result was not known, whilst 3 were discharged insane and 4 died.

The asylum is described as a palatial hotel, situated on the top of a hill, with commanding views. No special treatment is adopted beyond a total disuse of intoxicating liquors, and restraint within the boundaries of the establishment is only moral. The patients breakfast at 8 A.M.; attend prayers, and then amuse or employ themselves as they please till a one-o'clock dinner; supper is served at 6 P.M.; and the evenings are occupied with "readings," dramatic representations, or, one evening in the week, a lecture on temperance by the superintendent, Dr. Albert Day. No restriction is placed on the use of tobacco, and it is consumed in enormous quantities; coffee, also, is very largely consumed.

Permission to pass beyond the bounds of the asylum grounds can only be obtained from the superintendent, who judges of the amount of self-control acquired. Occasionally a "break-out" occurs, the patient usually returning at the end of his debauch, and being then placed in confinement for a day or two. Dr. Day insists upon the necessity for *total* abstinence after quitting the asylum; and maintains that "he has never known a man who had been intemperate to be able to drink at all without falling again into excess." We echo the wish of the writer in *Macmillan* that institutions of a kind similar to this asylum were established in this country.—*Lancet.*

